# Multi-Objective Control Optimization for Greenhouse Environment Using Evolutionary Algorithms

**DOI:** 10.3390/s110605792

**Published:** 2011-05-27

**Authors:** Haigen Hu, Lihong Xu, Ruihua Wei, Bingkun Zhu

**Affiliations:** 1 School of Information Engineering, Zhejiang Agriculture & Forestry University, Lin’an City 311300, Zhejiang Province, China; E-Mail: hnhhg@163.com; 2 Department of Control Science and Engineering, Tongji University, Shanghai 200092, China; E-Mails: laurrywei@sina.com (R.W.); zhu1981_2001@yahoo.com.cn (B.Z.)

**Keywords:** greenhouse environment control, PID control, feedback control, multi-objective optimization, evolutionary algorithms, nonlinear systems

## Abstract

This paper investigates the issue of tuning the Proportional Integral and Derivative (PID) controller parameters for a greenhouse climate control system using an Evolutionary Algorithm (EA) based on multiple performance measures such as good static-dynamic performance specifications and the smooth process of control. A model of nonlinear thermodynamic laws between numerous system variables affecting the greenhouse climate is formulated. The proposed tuning scheme is tested for greenhouse climate control by minimizing the integrated time square error (ITSE) and the control increment or rate in a simulation experiment. The results show that by tuning the gain parameters the controllers can achieve good control performance through step responses such as small overshoot, fast settling time, and less rise time and steady state error. Besides, it can be applied to tuning the system with different properties, such as strong interactions among variables, nonlinearities and conflicting performance criteria. The results implicate that it is a quite effective and promising tuning method using multi-objective optimization algorithms in the complex greenhouse production.

## 1. Introduction

The greenhouse environment control problem is to create a favorable environment for the crop in order to reach predetermined results for high yield, high quality and low costs. It is a very difficult control problem to implement in practice due to the complexity of the greenhouse environments. For example, they are highly nonlinear, strong coupled and Multi-Input Multi-Output (MIMO) systems, they present dynamic behaviors and they are largely perturbed by the outside weather (wind velocity, outside temperature and humidity, *etc.*) and also by many other practical constraints (actuators, moistening cycle, *etc.*). In recent years, various advanced control techniques and related strategies, such as predictive control [1–3], adaptive control [4], nonlinear feedback control [5], fuzzy control [6–8], robust control [9], optimal control [10,11] and compatible control [12] are widely proposed for different types of greenhouse environment control. These studies are important to real-world engineering application in greenhouse production. However, most of these approaches are either theoretically complex or difficult to implement in the actual greenhouse production, and the controller designs in the greenhouse engineering application mostly adopt the conventional proportional, integral, and derivative (PID) controllers owing to the simple architecture, easy implementation and excellent performance. Even so, they do not generally give an overall consideration of various properties, such as strong interactions among variables, nonlinearities, multiple constrains and conflicting objectives, that may exist in greenhouse climate control systems. Naturally, it is difficult for such controllers to achieve a satisfactory control effect, and the tuning of such controllers is still a challenge to process engineers and operators in the greenhouse production.

Furthermore, about 95% of the regulatory controllers of the process control, motor drives, automotive, fight control and instrumentation industries have PID structures. In spite of this widespread usage, the effectiveness is often limited owing to poor tuning, and tuning PID controllers efficiently is up to this time an interesting research. A lot of tuning methods have been presented in the extant literatures [13–16], which include designs based on guess-and-check (such as trial and error tuning method), linear control theory (such as Ziegler-Nichols (Z-N) and Cohen-Coon methods (C-C)), *etc.* Nevertheless, it is hard for these conventional tuning methods to achieve the desired performance of the controlled greenhouse because there is nearly no effective analytical way of finding the optimal set of gain parameters, and they are mostly based on linear models, which are usually adjusted around operating points. Empirical methods such as Z-N, which can be used for the tuning a simple problem, nearly fail to deal with the complex systems like greenhouse environment, owing to the lack of empirical data for a wide range of problems. Hence, new designs for tuning the PID parameters have to be explored to regulate the greenhouse environment.

Recently, an optimal tuning method of PID controller by employing Evolutionary Algorithms (EAs) has been proposed and successfully used in a wide range of plants. For example, Chang [13] proposed a modified crossover formula in genetic algorithms (GAs) to determine PID controller gains for multivariable processes. Arruda *et al.* [14] proposed an automatic tool to tune PID controllers in a MIMO process based on a Rank Niching Genetic Algorithm. The proposed scheme can be applied into a coupled MIMO process with several control loops, which are tuned in a unified way as a full MIMO controller. Herrero *et al.* [15] proposed a tuning scheme for robust PID controllers by adopting a specific multi-objective evolutionary algorithm. The scheme considers parametric uncertainty and can deal with model uncertainty by using a robust identification method. Ayala *et al.* [16] presented the design and the tuning of two PID controllers through Non-dominated Sorting Genetic Algorithm-II (NSGA-II) [17]. It is simple but effective to implement the robust solutions providing a good reference tracking performance in closed loop. These works mentioned above have obtained a certain effect by taking different approaches to different characteristics of their respective problems.

The main objective of this work is to develop an intelligent tuning method for greenhouse climate control with two PID loops of a MIMO process, which is characterized by strong interactions among process variables, nonlinearities and conflicting performance criteria. The problem is stated as a multi-objective optimization problem where the two PID controllers are simultaneously tuned based on different and possibly conflicting specifications, such as good static-dynamic performance, as smooth control signals as possible. The populations are encoded the gain parameters and the corresponding objective (cost) functions or fitness functions are formulated based on the specifications of disturbances rejections, static-dynamic performance and smooth control.

The rest of the paper is organized as follows. Section 2 describes the considered greenhouse climate model and the corresponding nonlinear differential equations. In Section 3, the PID controller structure is proposed based on performance criteria. Section 4 describes the multi-objective evolutionary algorithm used in this work. The simulations and results are presented in Section 5. Finally, a conclusion and prospects are given in Section 6.

## 2. Description and Problem Formulation

### 2.1. Greenhouse Climate Dynamic Model

The greenhouse environment is a complex dynamical system. Over the past decades, people have gained a considerable understanding of greenhouse climate dynamics, and many methods describing the dynamic process of greenhouse climate have been proposed. Traditionally, there are two different approaches to describe it; one is based on energy and mass flows equations describing the process [5,18–20], and the other is based on the analysis of input-output data from the process by using a system identification approach [21–23]. This paper deals with the first method for inside air temperature and humidity of a greenhouse, and its physical model describes the flow and mass transfers generated by the differences in energy and mass content between the inside and outside air [24]. Most of the analytic models on analysis and control of the environment inside greenhouses have been based on the following state space form:

$$\dot{x}=f(t,x,u,v)$$

where *x* are states variables like indoor temperature, humidity and carbon dioxide concentration, *u* are control inputs like energy input by the heating system, fogging systems, ventilation system and *CO*_2_ supply flux, *v* are external disturbances like solar radiation, outdoor temperature, humidity and wind speed, *t* denotes time, and *f*(·) is a nonlinear function.

**Figure 1.**
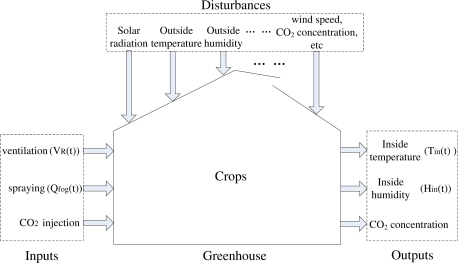
Greenhouse climate dynamic model.

In order to effectively validate the performance of the proposed algorithm below, the considered greenhouse analytic expression is based on the heating/cooling/ventilating model in this work, which can be obtained from many extant literatures [5,18]. It can be summarized in the functional block diagram given in Figure 1. Considering the associated high costs, *CO*_2_ supply systems do not have an extensive use, therefore the related variables are not taken into account in this work. To simplify the model, we consider only some primary disturbance variables, such as solar radiation, outside temperature and humidity. After normalizing the control variables, we consider the greenhouse dynamic model presented in [5] by using the following differential equations:

(1) $$\frac{{\text{dT}}_{\text{in}}(t)}{\text{dt}}=\frac{1}{C_{0}}[S_{i}(t)-\lambda^{\prime }Q_{\%,\text{fog}}(t)]-(\frac{V_{R,\%}(t)}{t_{v}}+\frac{UA}{C_{0}})\cdot [T_{\text{in}}(t)-T_{\text{out}}(t)]$$

(2) $$\frac{{\text{dH}}_{\text{in}}(t)}{dt}=\frac{Q_{\%,\text{fog}}(t)}{V^{\prime }}+\alpha^{\prime }S_{i}(t)-\frac{V_{R,\%}(t)}{t_{v}}\cdot [H_{\text{in}}(t)-H_{\text{out}}(t)]$$

where

*T_in_/T_out_* is the indoor/outdoor air temperature(°C),
*H_in_/ H_out_* is the interior/exterior humidity ratios (*g*[*H*_2_*O*]*kg^−^*^1^[dry air]),
*U A* is the heat transfer coefficient of enclosure(*W K^−^*^1^),
*C*_0_ = *ρC_p_V_TH_*, here *ρ* and *C_p_* are the air density (1.2 *kgm^−^*^3^) and the specific heat of air (1,006 *Jkg^−^*^1^*K^−^*^1^), respectively. *V_TH_* is the actively mixing air volume of temperature and humidity. Generally speaking, *V_TH_* is small as 60%–70% of the geometric volume *V* (*m*^3^) of the greenhouse.
*S_i_* is the intercepted solar radiant energy (*W*),
$\lambda^{\prime }=\lambda Q_{\text{fog}}^{\text{max}}$, here *λ* is the latent heat of vaporization (2257 *Jg^−^*^1^) and $Q_{\text{fog}}^{\text{max}}$ is the maximum water capacity of fog system (*gH*2*Os^−^*^1^),
*α*′ = *α*(*λV_H_*)*^−^*^1^, here *α* is scaling parameter, which is considered as constant over a short period due to its relatively low-frequency variation,
$V^{\prime }=V_{\text{TH}}/Q_{\text{fog}}^{\text{max}}$,
*t_v_* represents the time needed for one air change the sampling period.

The ventilation rate *V_R_* is measured as a percentage of the maximum ventilation rate $V_{R}^{\text{max}}$ (*i.e.*, $V_{R}=V_{R,\%}V_{R}^{\text{max}}$); Similar to *V_R,_*_%_, we define *Q*_%_*_,fog_* as a percentage of the maximum capacity of the fog system $Q_{\text{fog}}^{\text{max}}$.

### 2.2. Problem Formulation

The climate model provided above can be used in summer operation, and two variables have to be regulated, namely the indoor air temperature (*T_in_*) and the humidity ratio (*H_in_*), through the processes of ventilation (*V_R,_*_%_(*t*)) and fogging (*Q*_%_*_,fog_*(*t*)). The purposes of ventilation are to exhaust moist air and to replace it with outside fresh air, to regulate high temperatures caused by the influx of solar radiation, to dehumidify the greenhouse air when the humidity of the outside air is very low, to provide uniform air flow throughout the entire greenhouse, and to maintain acceptable levels of gas concentration in the greenhouse. Fogging systems (such as misters, fog units, or roof sprinklers) are primarily used for humidification of the greenhouse. In fact, fogging systems also play a cooling role due to evaporative cooling. Moreover, fresh air must be continually ventilated into the greenhouse while warmed and humidified air is exhausted. When humidifying is occurred under sunny conditions, ventilation is necessary since the greenhouse would soon become a steam bath without providing fresh dry air.

In order to effectively express the state-space form, we define the inside temperature and absolute humidity as the dynamic state variables, *x*_1_(*t*) and *x*_2_(*t*), respectively, the ventilation rate and the water capacity of the fog system as the control (actuator) variables, *u*_1_(*t*) and *u*_2_(*t*), respectively, and the intercepted solar radiant energy, the outside temperature, and the outside absolute humidity as the disturbances, *v_i_*(*t*), *i* = 1, 2, 3. Equations (1) and (2) can alternatively be written in the following state-space form:

(3) $${\dot{x}}_{1}(t)=-\frac{U\;A}{C_{0}}x_{1}(t)-\frac{1}{t_{v}}x_{1}(t)u_{1}(t)-\frac{\lambda^{\prime }}{C_{0}}u_{2}(t)+\frac{1}{C_{0}}v_{1}(t)+\frac{U\;A}{C_{0}}v_{2}(t)+\frac{1}{t_{v}}u_{1}(t)v_{2}(t)$$

(4) $${\dot{x}}_{2}(t)=-\frac{1}{t_{v}}x_{2}(t)u_{1}(t)+\frac{1}{V^{\prime }}u_{2}(t)+\alpha^{\prime }v_{1}(t)+\frac{1}{t_{v}}u_{1}(t)v_{3}(t)$$

Owing to the complexity appearing as the cross-product terms between control and disturbance variables, Equations (3) and (4) are obviously coupled nonlinear equations, which cannot be put into the rather familiar form of an affine analytic nonlinear system.

## 3. PID Controller Structure

A typical structure of a PID controller involves three separate elements: the proportional, integral and derivative values. The proportional value determines the reaction to the current error, the integral value determines the reaction based on the sum of recent errors, and the derivative value determines the reaction based on the rate at which the error has been changing. The mathematical description of its control law is generally written in the ideal form in (5) or in the parallel form in (6)

(5) $$u(t)=K_{p}(e(t)+\frac{1}{T_{i}}\int_{0}^{t}e(\tau )d\tau +T_{d}\frac{de(t)}{dt})$$

(6) $$u(t)=K_{p}e(t)+K_{i}\int_{0}^{t}e(\tau )d\tau +K_{d}\frac{de(t)}{dt}$$

where *K_p_* is the proportional gain, *T_i_* is the integral time constant, *T_d_* is the derivative time constant, *K_i_* = *K_p_/T_i_* is the integral gain and *K_d_* = *K_p_T_d_* is the derivative gain. And *e*(*t*) is the current error signal, which is defined as

(7) e(t)=r(t)−y(t)

where *r*(*t*) and *y*(*t*) are the reference signal and process output, respectively.

### 3.1. PID Controller for Greenhouse Climate System

**Figure 2.**
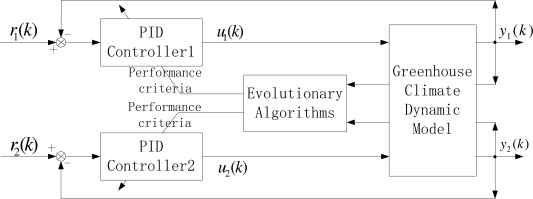
The diagram of a greenhouse climate control system.

Note that the greenhouse dynamic system mentioned above is a two-input and two-output continuous time nonlinear system. We consider a multi-variable PID control structure as shown in Figure 2. In order to simulate its behavior on a digital computer, we adopt a fourth-order Runge-Kutta method with a sufficiently small integration step, and we select the sampling time as a time step. Hence, considering a typical digital incremental PID control algorithm, the corresponding control law of each loop is given as:

(8) $$u(k)=u(k-1)+K_{p}(e(k)-e(k-1))+K_{i}e(k)+K_{d}(e(k)-2e(k-1)+e(k-2))$$

where *k* is iterative step.

### 3.2. Performance Criteria

Integral error is the most commonly used as a good measure for system performance. All kinds of such performance criteria, such as integrated absolute error (IAE), integrated square error (ISE), integrated time square error (ITSE) and integrated time absolute error (ITAE), are often employed in control system design. Killingsworth *et al.* [25] have demonstrated that ITSE can produce superior closed loop performance such as smallest overshoot, fastest settling time and less time required to initially reach the set point. It can represent well output specifications in the time domain. Hence, we consider the ITSE as one of performance criteria in this work, which is given as

(9) $$ITSE=\int_{0}^{\infty }te^{2}(t)dt$$

In addition, the performance criterion for one loop of MIMO process may be different from the other and some outputs may have the highest priority among the others. In this work, we only consider the same priority for the same class of performance criteria. Consequently, to be convenient for a digital simulation of the system, the performance index in (9) for the MIMO system in the greenhouse is rewritten as follows:

(10) $$J_{1}=\sum_{k=1}^{\infty }(t(k)\sum_{i=1}^{2}e_{i}^{2}(k))$$

where *i* is the *i^th^* close loop, *t*(*k*) represents the time of the *k^th^* iterative.

Another aspect, the seriously oscillatory of control signals can do great damage to the actuators, and it is not acceptable in the design of controllers. Therefore, to avoid such case and perform a smoothing operation, we consider another performance index defined as

(11) $$J_{2}=\sum_{k=1}^{\infty }\frac{1}{2}\Delta u^{2}(k)=\sum_{k=1}^{\infty }\frac{1}{2}{\left(u(k)-u(k-1)\right)}^{2}$$

It is worthy to notice that minimizing the first performance index *J*_1_ will provide good static-dynamic performance and better disturbance rejection, while minimizing the second *J*_2_ will perform a smoothing operation and avoid the serious oscillating of the actuators.

## 4. Multi-Objective Evolutionary Algorithms

Evolutionary algorithms simulate the survival of the fittest in biological evolution by means of algorithms, and they are becoming increasingly valuable in solving real-world engineering problems. Compared with single-objective evolutionary algorithms, multi-objective techniques have many advantages, especially for the problem with multiple conflicting objectives. For example, they can search for a set of solutions with different trade-offs from a family of equivalent solutions, which are superior to other solutions and are considered equal from the perspective of simultaneous optimization of multiple competing objective functions. Such solutions are generally called non-inferior, non-dominated or Pareto optimal solutions.

The design of the controller usually need to satisfy multiple performance requirements such as good static-dynamic performance and smoothing control operation. However, it is almost impossible to attend the above requirements simultaneously. For instance, in many cases, seeking for some dynamic specifications usually causes the large variance of control law and the serious oscillating of the actuators. Consequently, a satisfactory tradeoff must be found and a set of optimal solutions must be provided by minimizing the performance index *J*_1_ and *J*_2_. That is, *J*_1_ and *J*_2_ are taken as two objective (cost) functions or fitness functions for the optimization process.

Multi-objective optimization methods are used to obtain Pareto solutions for multiple conflicting objectives. Many algorithms, such as NSGA-II [17], Strength Pareto Evolutionary Approach 2 (SPEA2) [26] and the Pareto Archived Evolution Strategy (PAES) [27], appear to be very promising ways to approximate Pareto fronts. Considering newly developed and versatile multi-objective evolutionary algorithms, we adopt NSGA-II to optimize the performance criteria in this work, because it is a computationally efficient algorithm implementing the idea of a selection method based on classes of dominance of all the solutions. Each individual *ind* in the evolutionary algorithm represents the gain parameters of PID controllers. Therefore, the problem can be formulated as follows:

Find *ind* = [*K_p_*_1_, *K_i_*_1_, *K_d_*_1_, *K_p_*_2_, *K_i_*_2_, *K_d_*_2_]
to optimize *J*_1_(*ind*, *u*), *J*_2_(*ind*, *u*)
subject to *a_i_* ≤ *ind*(*i*) ≤ *b_i_*, *c_j_* ≤ *u_j_* ≤ *d_j_*
for *i* = 1,..., 6, *j* = 1, 2

where *a_i_* and *b_i_* are bound constraints of the gain parameters, which are chosen by trial and error simulation experiments. *c_j_* and *d_j_* are bound constraints of the control inputs.

The specifications in the time domain, such as overshoot, rise time, settling time and steady-state error, are incorporated into NSGA-II and are calculated at each iteration, just the same as the objective functions (*i.e.*, *J*_1_ and *J*_2_) of the optimization process. When the obtained performance measures are infeasible during the process of step response, for example, there are not enough data to find rise time (*t_r_*) and settling time (*t_s_*), that is to say, the system is unstable, we formulate the penalty for the objective functions *J*_1_ and *J*_2_ as follows:

$$J_{1}=\{\begin{array}{llll} \infty , & \text{if}\;t_{s}==\infty & \left|\right| & t_{r}==\infty ,\\ J_{1}, & \text{otherwise}, & & \end{array}$$

$$J_{2}=\{\begin{array}{llll} \infty , & \text{if}\;t_{s}==\infty & \left|\right| & t_{r}==\infty ,\\ J_{2}, & \text{otherwise}, & & \end{array}$$

where the performance measures *t_r_* and *t_s_* are given with infinity, respectively, when they are not found. Therefore, the infeasible solutions are immediately discarded upon creation, and not considered further during evolution, owing to the infinity of objective functions. Conversely, there exist no influence on the normal situation.

## 5. Simulations and Results

In the present section, a simulation experiment is presented to demonstrate the validity of the proposed method. For this example, we consider a greenhouse of surface area 1,000 *m*^2^ and a height of 4 *m*. The greenhouse has a shading screen that reduces the incident solar radiation energy by 60%. The maximum water capacity of the fogging system is 26 *g*[*H*2*O*]*min^−^*^1^*m^−^*^3^. The maximum ventilation rate corresponds to 20 air changes per hour.

**Figure 3.**
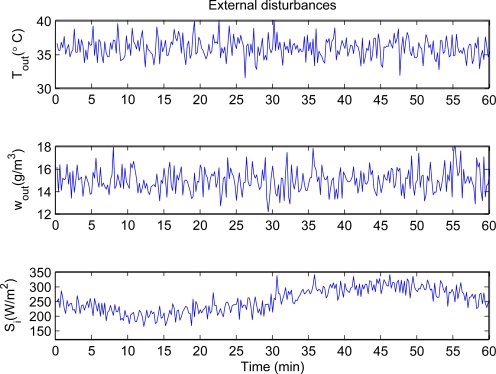
Changes of outdoor air temperature, humidity ratio and solar radiation.

The active mixing air volume of the temperature and humidity is given as *V_TH_* = 0.65 *V*. The greenhouse model parameters (shown in Table 1) are presented by [5] through identifying method, which are expressed per square meter (*m*^2^) of greenhouse area. Moreover, the initial values of indoor air temperature and humidity ratio are 32 °C and 12 g[H2O]/kg[air], respectively. The set points of indoor air temperature and humidity ratio are 25 °C and 21 g[H2O]/kg[air](the corresponding relative humidity about 70%), respectively. The external disturbances are shown in Figure 3. We consider the real-coded NSGA-II for the optimization. The corresponding operators and parameters are shown in Table 2. Simulations are performed under the environment of Matlab R2010a (version 7.10.0.499) and Microsoft Windows XP Professional SP2 OS. Computer used for these simulations is Intel(R) Core(TM)2 Duo CPU, 1.83 GHz, 2 GB of RAM.

**Figure 4.**
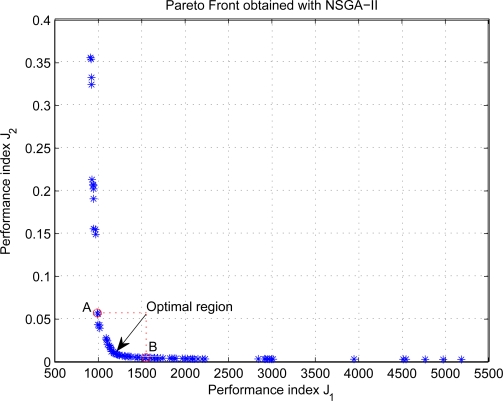
Pareto Front of performance index *J*_1_ and *J*_2_.

Step signals are given for the inputs of controllers, and it takes about 11 minutes for each simulation. Figure 4 shows the Pareto front of simulation results using NSGA-II, and the results indicate that the performance objectives are conflicting decision criteria with each other. As can be seen from the Figure, the value of the index *J*_2_ drops sharply as that of the index *J*_1_ increases on the left of point *A*, whereas the index *J*_2_ remains almost unchanged with the increase of the index *J*_1_ on the right of point *B*. It suggests the Pareto Front *AB* is the optimal desired region for most situations. Therefore, a satisfactory tradeoff may be found from the region, and Decision Maker (DM) can select the best final solution according to his or her preference.

**Figure 5.**
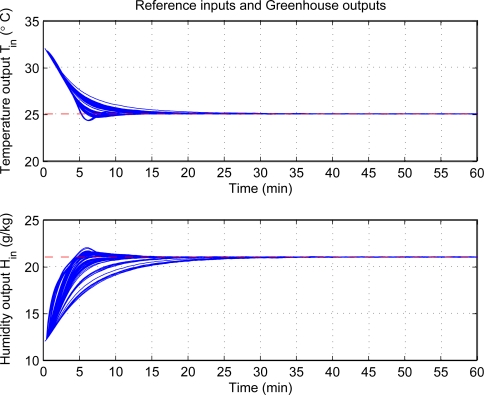
Step responses with PID control at each individual.

**Figure 6.**
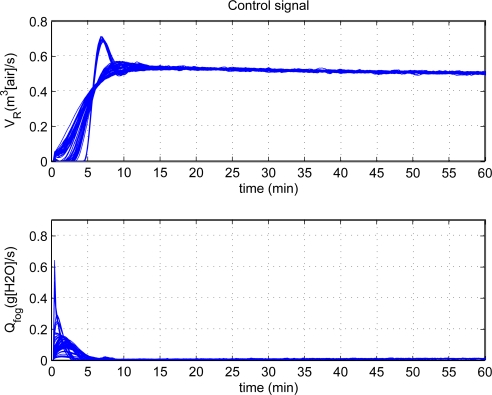
The corresponding control signals.

Figure 5 shows the effectiveness of step responses with PID control at each individual. The top plot shows the response from 32 °C to 25 °C for the inside air temperature, and the curve distribution is centralized and it reveal similar responses for each individual of population. The bottom plot shows the humidity change from 12 *k/kg* to 21 *k/kg*, and the rising time and setting time of some responses are longer than others and it shows the different response characters. Besides, both figures show the steady-state error is very small and reveal that they have good steady state performance. Moreover, the tuning scheme can achieve very good control performance as a whole. The corresponding control signals are illustrated in Figure 6. There are hardly oscillating process and the control signals are smooth. To achieve the desired climatic condition, the fogging systems should operate at high power, while low power for ventilation systems in the initial stage of control process. Then, the fogging systems maintain low power to operate, while high power for ventilation systems.

**Figure 7.**
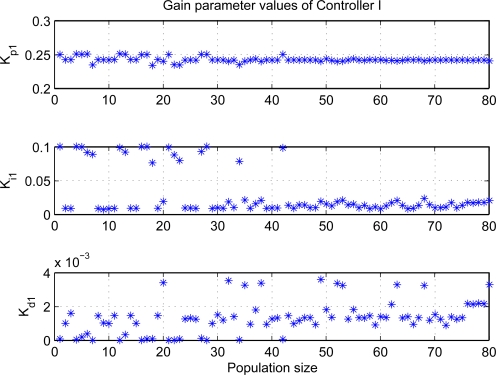
PID gain parameters of 1*^th^* loop at each individual.

**Figure 8.**
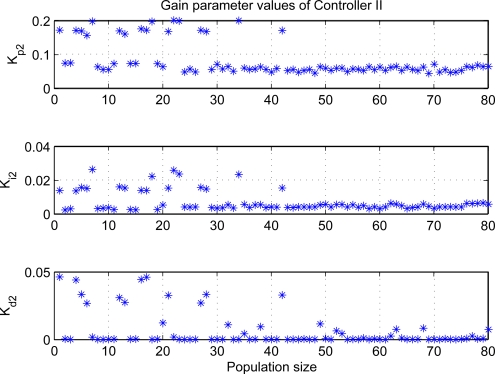
PID gain parameters of 2*^th^* loop at each individual.

Figures 7 and 8 show the distributions of PID gain parameters at each individual. From the results, the controller I has small derivative gain, or even it may be implemented by adopting PI loop under the most conditions, whereas the proportional gains are large for two control loops. Moreover, the gain parameters tend to the same value with the evolution process, which reveals evolution completely during optimization processes and can find the optimal gain parameters.

In order to demonstrate the effectiveness of the proposed tuning method, we consider other performance criteria such as overshoot, rise time, settling time and steady-state error. For simplification of analysis, a simple mean for the respective type performance criteria at each individual is implemented. Table 3 shows the maximum, minimum, mean and standard deviation of these specifications in the time domain. As can be seen, the maximum values of overshoot, rise time, settling time and steady state error are 3.6475%, 11.0482 minutes, 15.4252 minutes and 0.0285, respectively. Compared with other systems, the rise time and settling time are large, but the values are small enough for greenhouse production. It is worth notice that the values of minimum, mean and standard deviation are 0.0618%, 0.998% and 1.0429% in the column of overshoot, respectively. Nevertheless, there exist many curves without overshoot in the two responses of Figure 5, which implies that at least one of the two loops has overshoot. On the whole, these specifications shown in Table 3 reveal the system has good static-dynamic performance, and are completely acceptable for greenhouse climate control system.

## 6. Conclusions

PID controllers have been extensively used in the greenhouse production process owing to their simple architecture, easy implementation and excellent performance. However, the tuning of several controllers in the complex greenhouse environment is a challenge to process engineers and operators. Many controllers are poorly tuned in practice due to the complexity of the controlled greenhouse such as the dynamical behavior of greenhouse climate and control requirements, which present strong interactions among variables, non-linearities, multiple constrains and conflicting objectives.

This paper presented the tuning of two PID controllers through NSGA-II based on multiple performance measures such as good static-dynamic performance and smooth control signals. The proposed tuning scheme has been tested for greenhouse climate control by minimizing ITSE and control increment or rate in a simulation experiment. Results show the effectiveness and usability of the proposed method for step responses. The obtained gains are applied in PID controllers and can achieve good control performance such as small overshoot, fast settling time, and less rise time and steady state error.

The results suggest that the proposed tuning scheme using multi-objective optimization algorithms is a quite promising method and it presents the following features: (i) it can be applied in the cases that the empirical methods fail to be used; (ii) it can effectively solve the strong interactions among process variables; (iii) it can be applied into certain strong nonlinear control system including nonconvex problems due to adopting global optimization algorithms.

It should be noted that this study has only examined the analytic greenhouse model. We have to point out that it is not suitable using such an approach if an analytic model is not provided, because there is nearly no effective way to formulate the objective (cost) functions or fitness functions of evolutionary algorithms required. Besides, this approach is time-consuming and heavily dependent on the computation time. It is not suitable for an online real time control requirement. Not withstanding its limitation, this study does suggest that the online optimal operation for greenhouse production process will be further studies.

Certainly, the method is not limited to greenhouse applications, but could easily be extended to other applications, and we expect that it will become more widely used in the future for other types of systems and controllers.

## Figures and Tables

**Table 1.** Identified greenhouse model parameters.

Parameters name	unit expression	values
*C*_0_	*minW°*C*^−1^*	−324.67
*U A*	*W°*C*^−1^*	29.81
*t_v_*	*min*	3.41
*λ*′	*W*	465
*α*′	*gm^−3^min^−1^W^−1^*	0.0033
1/*V*′	*gm^−3^min^−1^*	13.3

**Table 2.** Operators and parameters of real-coded NSGA-II.

Description	values
Population size	80
Number of generations	50
Probability of crossover of real variable	0.9
Probability of mutation of real variable	0.5
Distribution index for crossover	10
Distribution index for mutation	20
Lower limits of the gain parameters(*i.e.*, *a_i_*)	[0, 0, 0, 0, 0, 0]
Upper limits of the gain parameters(*i.e.*, *b_i_*)	[0.5,0.1,0.1,0.2,0.1,0.1]
Lower limits of the control inputs(*i.e.*, *c_j_*)	[0, 0]
Upper limits of the control inputs(*i.e.*, *d_j_*)	[1, 1]
Sampling time (min)	0.2

**Table 3.** The corresponding performance criteria (mean of two loops).

Performance criteria	Overshoot (%)	Rise time (min)	Settling time (min)	Steady-state error
Description				
Maximum	3.6475	11.0482	15.4252	0.0285
Minimum	0.0618	3.2781	4.6522	0.0018
Mean	0.9980	5.2858	7.6943	0.0110
Standard deviation	1.0429	2.1129	2.9504	0.0054
